# Histological and Serological Epidemiology of Hepatitis Delta Virus Coinfection among Patients with Chronic Active Hepatitis B Virus in Razavi Khorasan Province, Northeastern Iran

**Published:** 2018-12

**Authors:** Payam SHARIFAN, Sakineh AMOUEIAN

**Affiliations:** Dept. of Pathology, School of Medicine, Mashhad University of Medical Sciences, Mashhad, Iran

**Keywords:** Hepatitis delta virus, Chronic active hepatitis B virus, Coinfection, Seroprevalence, Iran

## Abstract

**Background:**

Hepatitis delta virus (HDV), as well as hepatitis B virus (HBV), are regarded as one of the main public health issues in developing countries. This retrospective study described histological and serological features of HDV coinfection patients with chronic active HBV in Northeastern Iran.

**Methods:**

The frequency of HDV seropositivity and its impact on serum liver enzyme levels and pathological features were investigated by reviewing clinical and laboratory data. This study contained chronic active HBV-infected patients having admitted the department during 2009 and 2014.

**Results:**

The rate of HDV coinfection in chronic active carriers was 21.84%, with a male predominance. HDV seropositive carriers showed significantly higher concentrations of liver enzyme than chronic active HBV monoinfection. Moreover, there was a strong association between degrees of inflammation with HDV-positive patients’ enzyme levels.

**Conclusion:**

The HDV seroprevalence in northeastern Iran was higher than that reported from elsewhere in Iran while comparable to some regions in Middle East, which, in turn, requires more comprehensive tools for diagnosing and screening the blood.

## Introduction

Hepatitis delta virus (HDV) as a defective RNA-virus is more likely to induce severe acute and chronic liver complications in those people afflicted with hepatitis B virus (HBV). HDV depends on HBV due to lack of its own polymerase during propagation (1); put it differently, HDV genome is closely connected with an HBV outer lipoprotein layer (three HBV envelope proteins and host lipids) (2). HBV as a human-specific virus is a member of the hepadnaviridae family, having a peculiar genome structure and replication cycle (3). An estimation of above 15 million people chronically have found to be infected with HDV all the world over (4). Five percent of total already infected with HBV communicate HDV, as well (5). On the other hand, 240 million were hepatitis B surface antigen (HBsAg) positive chronic carriers according to the WHO (6). In Iran, almost 1.7% of the general population has had chronic HBV infection (7). As for its mortality, around 780,000 people die per year because of HBV infection (8).

There are two main types of HDV infection, namely coinfections as well as superinfections. A coinfection may appear acute and asymptomatic, present multiple common symptoms including fatigue, lethargy, anorexia, and nausea, or come up with acute liver failure (9). Conversely, an HDV super-infection is chronically developed in most cases, characterized by liver inflammation, fibrosis, and decompensated liver cirrhosis (10, 11). In other words, it is largely correlated to severe liver condition, leading to cirrhosis in majority of patients (80%) (12). Therefore, the likelihood of end-stage liver disease and hepatocellular carcinoma increases in these cases more than HBV monoinfections (13).

Several studies specify endemic areas in Eastern and Southern Europe, Central and Eastern Africa, the Amazon Basin, parts of Asia and the Middle East (4). The epidemiologic profile of HDV infection differs from country to country worldwide. Iran, akin to USA, has various regions (14), each of which shows disparities in HDV prevalence. HBV/HDV coinfections occurred in 5.7% in Tehran (14). An approximately similar prevalence was reported in northeastern Iran (15). On the contrary, 2.4% were infected with HDV and HBV in west of Iran (16). Interestingly, 8.8% of HBsAg carriers also presented anti-HDV in Tehran (17). Anti-HDV was found amongst 2.5% of patients with asymptomatic chronic HBsAg. This hovered around 49.2% in HBsAg positive cases who had chronic active hepatitis and cirrhosis (16). In Shiraz, hepatitis D infection consisted 9.3% of individuals communicating chronic active hepatitis B (18).

This study aimed at investigating the serum functional liver enzyme as well as pathological features in chronic active HBV patients with and without HDV coinfection in the northeastern part of Iran.

## Materials and Methods

The present investigation was designed as a single-center and retrospective study to analyze the influence of HDV infection on chronic active hepatitis B-associated histopathology of the liver. This study was approved by Medical Research Committee, Mashhad University of Medical Sciences.

Patients with chronic active hepatitis B on liver biopsy were selected from Hepatology Department of Imam Reza Hospital, Mashhad, Iran from Apr 2009 to Mar 2014. They were then assigned into Groups A and B. Eligibility criteria at the time of assessment included positive serum HBsAg and anti-HDV as well as baseline level of functional liver enzymes higher than normal levels of serum glutamic oxaloacetic transaminase (AST) and serum glutamic pyruvic transaminase (ALT). These eligible patients were considered as Group A. Participants with seropositive for HBsAg yet seronegative for anti-HDV served as Group B. The measures of total anti-HDV (IgG and IgM) were obtained employing one step competitive enzyme-linked immunosorbent assay (ELISA; Dia Pro Diagnostic Bioprobes, Srl, Italy) kits. Those cases with a history of alcohol abuse, hepatocellular carcinoma, hepatitis C virus, human immunodeficiency virus, autoimmune or hereditary liver disease were excluded from the primary recruitment. Additionally, there was no female patient who was pregnant or lactating. The serum liver enzymes (ALT and AST aminotransferases) and liver histopathology (grade, stage, and total score of liver disease) were retrieved from the medical records and compared between the two groups. The histopathologic severity was determined for inflammation (grade) and stage by considering Scheuer classification (19). Data were collected and fed into SPSS (ver. 11.5, Chicago, IL, USA). Independent Samples t-test, Mann Whitney U test or chi-square test was used to examine statistically significant differences between the two groups’ characteristics. The correlations between these variables were also performed through Pearson for normally distributed data. All analyses were significant at *P*<0.05.

## Results

This study contained a number of 87 participants with chronic active HBV. Of 19 were considered in Group A and 68 patients were only HDV-negative (Group B). Both groups were comparable across the distribution of sex and age (*P*>0.05). The mean age was 41.26±10.87 yr (Table 1). Approximately half of each group was women. Although the mean ALT and AST for Group A was higher than their counterparts in Group B, there were no significant differences between two groups in serum level of ALT and AST (*P*>0.05). The mean scores of stage and grade in Group A were 2.74±1.52 and 7.37±2.19 which declined in Group B to 1.69±1.74 and 6.59±3.23, respectively. Both groups were approximately similar for grade of inflammation (*P*=0.155) whereas remarkably disparate in stage of fibrosis (*P*=0.005). The mean total score (10.05±3.15 vs. 8.28±4.58, *P*=0.047) was considerably higher in Group A.

**Table 1:** Baseline features in HDV positive and negative carriers

***Variable***	***Group A*** ***N=19***	***Group B*** ***N=68***	***P-value***
Age (yr) (mean ±SD)	45.58±11.79	40.06±10.37	0.076
Female, N (%)	7(8.05)	20(22.99)	0.052
AST, IU/L (mean ±SD)	65.68±40.24	46.01±33.14	0.062
ALT, IU/L (mean ±SD)	70.00±46.85	61.94±55.51	0.529
Stage (mean ±SD)	2.74±1.52	1.69±1.74	0.005
Grade (mean ±SD)	7.37±2.19	6.59±3.23	0.155
Total score (mean ±SD)	10.05±3.15	8.28±4.58	0.047

Overall, the histopathologic scores were strongly associated with the levels of functional liver enzyme in Group A. There was a significantly strong relationship of total score and inflammation with the mean concentrations of ALT and AST with (r>0.5, *P*<0.05). The mean ALT and AST had a moderate correlation with fibrosis in chronic active HBV patients coinfected with HDV (0.346 vs. 0.330; *P*>0.05). As for Group B (chronic active HBV monoinfection), the mean AST had a positively moderate correlation with stage, grade, and total score among HDV-negative patients (r<0.5, *P*<0.05). The mean ALT concentration failed to show a comparatively stronger correlation with the histopathologic severity than the mean AST (Table 2).

**Table 2:** Correlation between histological severity and liver enzyme level

***Variable***	***Stage***			***Grade***			***Total score***		
	***Group A***	***Group B***	***All patients***	***Group A***	***Group B***	***All patients***	***Group A***	***Group B***	***All patients***
AST	0.346	0.437^*^	0.446^*^	0.586^*^	0.334^*^	0.383^*^	0.576^*^	0.402^*^	0.445^*^
ALT	0.330	0.308^*^	0.317^*^	0.620^*^	0.165	0.230^*^	0.603^*^	0.234	0.289^*^

## Discussion

There are a lot of studies which address HDV seropositivity amongst Iranian patients (18, 20). However, very few efforts have been made concerning chronic active HBV carriers coinfected with HDV. This retrospective study was performed to measure the seroprevalence of HDV infection in chronic active HBV subjects referred to Hepatology Department of Imam Reza Hospital, Mashhad, Iran. This is the first report from this region as the largest referral center in northern Iran. Of 87 subjects who were positive for HBsAg, the frequency of HDV antibody was 21.84% which was a far beyond those reported from other parts of Iran (9.7%) (18). Akin to our findings, 20.8%, and 27.5% were substantiated for chronic active HBV coinfected with HDV in Iraq and Turkey, in order (21, 22). In the same setting, 6.6% of patients were reported with chronic active HBV positive for anti-HDV IgM in Iraq. Of note, no case of positive IgG was documented (23). However, Zahedan in the southeastern part of Iran has virtually close HDV seroprevalence around 17% throughout 2008 and 2011 (24). On the other hand, in contradiction with the survey in Hamedan (25), a considerably higher rate of HDV seroprevalence was found, reaching 17.3% among chronic HBV subjects referred to Hepatitis community center of Hamedan (20). In this regard, 14.8% showed positive results for both HDV RNA and anti-HDV with the concomitant infection of chronic HBV (26). HDV seroprevalence among chronic HBV patients varies in countries. For example, Cameron and Nigeria present the frequency of HDV seropositivity around 17.6% and 15%, respectively (27, 28). This disparity is highlighted in the reports from India as 5–10%, Thailand as 0.7% and Bangladesh 24.4% (29–31). Iran presented the great disparity from 2% in Qom and Babol to 20.7% in Kerman (32–34). When it comes to countries bordering Iran, Turkey, Iraq, Pakistan, Iraq, and Saudi Arabia shows the decreasing order of seroprevalence rate: 45.5%, 16.6%, 16.6%, and 3.3% (21, 35–37).

Age and sex did not affect anti-HDV antibody seropositivity status amongst HBV patients. Likewise, some scholars have reported that there is no relationship between sex and HDV positive status (15, 38, 39). Conversely, age has been shown to be an influential correlate of HDV infection (38, 40). As opposed to another study, HDV seropositivity prevails among males (15). The enzyme level was higher in HDV seropositive carriers, corroborated by previous reports (18, 38, 40). This was also evident in surveys from other parts of the world (41), explained in a way that HDV infection can afford to increase the inflammation of liver. Furthermore, chronic HDV-HBV coinfection leads to more severe liver disease than chronic HBV monoinfection (42, 43). Nevertheless, Ataei et al. did not observe such changes in serum enzyme level (39).

Histological assessment of the participants with coinfection of HDV and chronic active HBV demonstrated that mean stage of fibrosis and mean grade of inflammation were significantly greater in comparison with HBV alone. There is more severe hepatitis in HBV/HDV superinfection rather than HBV alone (44, 45). Besides, patients with HBV/HDV coinfection exhibit the higher likelihood for decompensated cirrhosis and mortality when compared with HDV negative cases (46). HDV prevalence in the Asia-Pacific region has been reduced (47); however, they came to this conclusion by using only reports from the late 1990s. This was in sharp contrast with the recent evidence from this region, namely Pakistan and South Korea where contained a high rate of HDV/HBV coinfection (48–50).

Serological analysis indicated that mean ALT and AST values were highly associated with stage of fibrosis and grade of inflammation, with Group A showing stronger strength. Similarly, patients afflicted with HBV and HDV have greater ALT concentrations and accordingly more fibrosis values (51). A cirrhosis frequency of 4% was found and HCC frequency of 2.8% amongst patients with persistent HDV infection (13). The histological as well as serological findings of the present study put stress on the previous results that HBV/HDV coinfection is more likely to increase the severity of liver disease (52). The undesirable potential effects of HDV on HBV management were also indicated in a mathematical model (53).

These studies along with our study showed that the HDV seroprevalence is on the rise in spite of applying HBV vaccine and examining the blood for blood-borne pathogens (54–56). This trend is not limited only to developing countries yet requires more attention even in industrial nations such as UK and Germany (57–59). Considering globalization as well as unprecedented increase in migration rate, it necessitates the significance of identifying and screening high-risk groups by using a comprehensive policy.

## Conclusion

The findings of the present study exhibited that northeastern Iran appeared to a high prevalent area, the same as some neighboring countries in Middle East. The HBV/HDV coinfection might be associated with the increased severity of liver disease.

## Ethical considerations

Ethical issues (Including plagiarism, informed consent, misconduct, data fabrication and/or falsification, double publication and/or submission, redundancy, etc.) have been completely observed by the authors.
